# Regulation of proliferation and cell cycle by protein regulator of cytokinesis 1 in oral squamous cell carcinoma

**DOI:** 10.1038/s41419-018-0618-6

**Published:** 2018-05-11

**Authors:** Fanglong Wu, Xueke Shi, Rui Zhang, Yuan Tian, Xiangjian Wang, Changlei Wei, Duo Li, Xiaoyu Li, Xiangli Kong, Yurong Liu, Weihua Guo, Yiqing Guo, Hongmei Zhou

**Affiliations:** 1 State Key Laboratory of Oral Diseases, Department of Oral Medicine, West China Hospital of Stomatology, Sichuan University, 610041 Chengdu, Sichuan China; 2 Department of Preventive Dentistry, West China Hospital of Stomatology, Sichuan University, 610041 Chengdu, Sichuan China; 3 State Key Laboratory of Oral Diseases, West China Hospital of Stomatology, Sichuan University, 610041 Chengdu, Sichuan China; 4 Department of Pediatric Dentistry, West China Hospital of Stomatology, Sichuan University, 610041 Chengdu, Sichuan China; 5 Department of Stomatology, The Affiliated Hospital of Qingdao University, 266003 Qingdao, Shandong China

## Abstract

Protein regulator of cytokinesis 1 (PRC1), a microtubule-associated protein, has emerged as a critical regulator of proliferation and apoptosis, acting predominantly in numerous tumors. However, its function in oral squamous cell carcinoma (OSCC) is still unknown. To establish the roles of PRC1 in OSCC, 95 oral clinical samples (54 OSCC, 24 oral leukoplakia [OLK], and 17 normal oral mucosa) and seven oral cell lines (6 OSCC and 1 normal oral cell lines) were analyzed using a series of molecular and genomic assays both in vivo and in vitro were conducted in this study. Herein, we provide evidence demonstrating that expression of PRC1 closely correlates with the degree of epithelial dysplasia in OLK (*n* = 24) (*p* *<* 0.001), and the poor differentiation, large tumor volume, lymph node metastasis, and high-clinical stage in OSCC (*n* = 54) (*p* *<* 0.05), illustrating that PRC1 has a promotive influence on tumor progression in OSCC. Simultaneously, we observed that PRC1 knockdown in OSCC cell lines caused G2/M phase arrest (*p* *<* 0.05), inhibited cell proliferation in vitro (*p* *<* 0.05) and tumor growth in vivo (*p* *<* 0.001). Furthermore, the effects of PRC1 on the regulation of proliferation and cell cycle transition in OSCC samples were mediated by p53. The p53/PRC1/EGFR signaling pathway was found to be implicated in the tumor progression of OSCC. Based on our data, we demonstrate that PRC1 is a key factor in regulating proliferation and the cell cycle, pointing to the potential benefits of PRC1-targeted therapies for OSCC.

## Introduction

Oral cancer, including oropharyngeal cancer, is, overall, the sixth most common cancer^1^. An estimated 300,400 new cases and 145,400 deaths occur globally from oral cavity cancer each year, and ample evidence indicates that the incidence of oral cancer is rising in many countries^2–4^. Generally, surgical resection, which may or may not be associated with radio and chemotherapy are still the gold standard for treatment. However, the 5-year overall survival is still about 50% and the prognosis depends principally on early detection and probable therapeutic approaches^3^. For the early staged tumors, the cure rate is an excellent nearly 95% with a rate of local recurrence rate of less than 5%, while the cure rate drops to 20–35% for advanced tumors with frequent recurrence and regional lymph node metastasis^5–7^. Thus, to seek less drug toxicity and obtain better clinical efficacy, the novel targets for the improvement of the patients’ quality life is increasingly required.

Molecularly targeted therapy has been endorsed with the aim of guiding personalized treatment by modulating discrete molecular functions that are specific to cancer cells and not the non-malignant tissue^8^. To date, some of the molecularly targeted agents have already been programmed in the clinic with favorable clinical outcomes^9,10^. However, for many molecularly targeted agents, there remains controversy regarding their efficacy and safety. The most controversial aspect of treatment design is the difficultly locating the “Achilles’ heel” in thousands of mutant genes and proteins which regulates tumor progression. Using a systems biology approach, the discovery of disease-related genes, proteins, and prognostic biomarkers for cancer treatment can be achieved systematically by studying whole crosstalk among the genes or proteins^11–13^. In our preliminary study, we have successfully developed a systems biology strategy that combines experimental and computational analyses for prediction of epithelial targets in an interactive network between proteins^12^. Based on these, we have forecasted that protein regulator of cytokinesis 1 (PRC1) is one of the potential targets for the treatment of oral cancer ^12^.

PRC1, also known as anaphase spindle elongation 1 (ASE1), belongs to the microtubule-associated proteins superfamily^14^. It regulates the proliferation and cytokinesis through crosslinking microtubules, with its crosslinks dynamically tracking antiparallel microtubule overlap^15,16^. So far, given studies have demonstrated that its abnormal expression has a vital role in the proliferation and apoptosis in many tumor types, including breast and bladder cancers^17,18^. Furthermore, its interconnections can also be seen in numerous other proteins such as CCDC69, KIF14, CLASP1, and p53^19–22^. However, only Hela and 293T cell lines have been studied in this research, with no exploration in head and neck cancer, especially in oral cancer. Accordingly, we speculate that PRC1 might regulate tumor progression in oral squamous cell carcinoma (OSCC). In the present study, we have verified the function and identified potential mechanisms of PRC1 in OSCC through in vivo and in vitro assessments.

## Results

### Overexpression of PRC1 in human clinical samples and cell lines

**Fig. 1:**
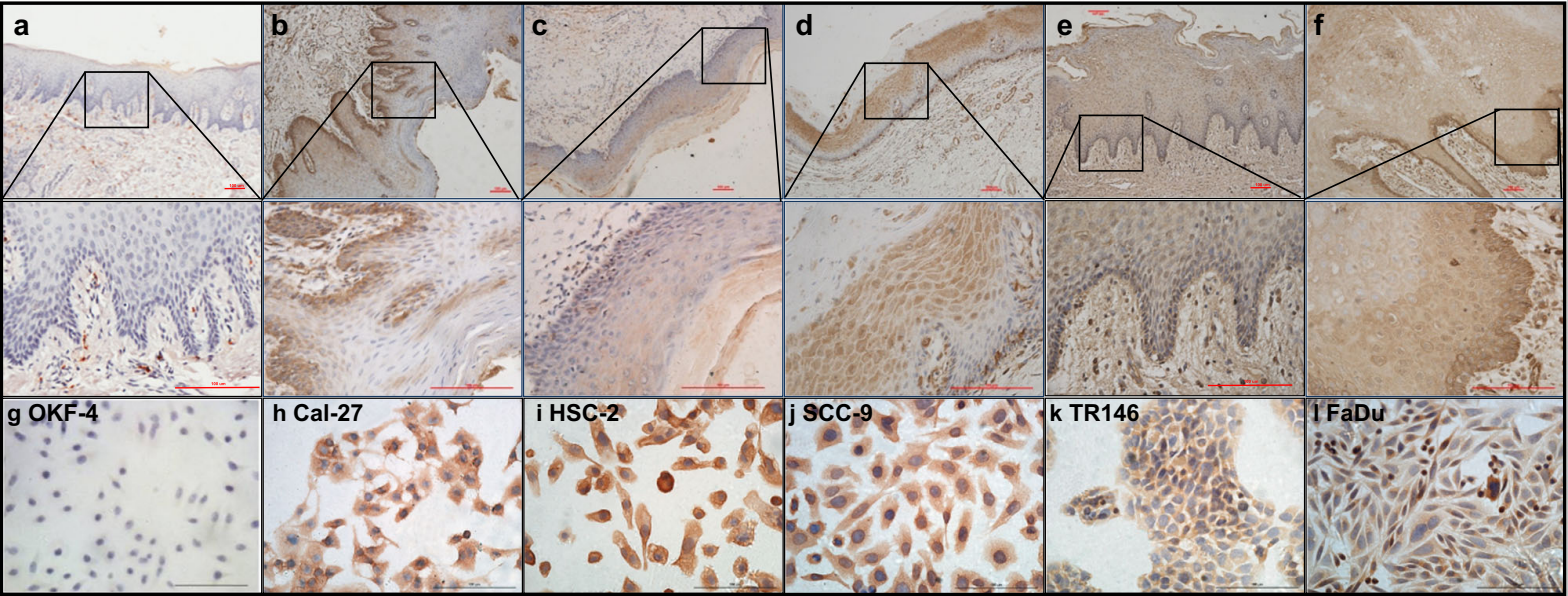
PRC1 expression is shown in normal epithelium, OLK, and OSCC. **a** PRC1 was not detected in the normal epithelium. **b** In the epithelium of OLK with no dysplasia, IHC demonstrated weak positive expression of PRC1, mainly located in the basal layer cells. **c** OLK with mild dysplasia exhibited weak positive expression of PRC1, mainly located in the stratum spinosum cells. **d** OLK with moderate to severe dysplasia expressed PRC1 in both basal layer and stratum spinosum cells. **e** Positive expression of PRC1 was observed in highly differentiated OSCC. **f** In the presence of poorly differentiated OSCC, diffuse positive and strongly positive cells were observed in epithelium. **g** PRC1 expression was negative or weak positive in OKF-4 cells. **h**–**l** OSCC cell lines exhibited positive staining commonly observed in OSCC, mainly located in the cytoplasm. Scale bar = 100 μm

Firstly, we evaluated the expression of PRC1 in 24 oral leukoplakia (OLK) and 54 OSCC specimens (Fig. 1), which represent different clinicopathologic stages (Tables 1 and 2). In normal oral tissues, the level of PRC1 was almost undetectable or had weakly positive staining (Fig. 1a). However, overexpressed PRC1 in OLK and OSCC was detected (Fig. 1b–f), and the difference was statistically significant when compared to normal oral mucosa (*p*_OLK_ = 0.000, *p*_OSCC_ = 0.000). A comparison of OLK and OSCC demonstrated that PRC1 was expressed in different cellular locations (Fig. 1b–f). In addition, the positive rate and staining score were upregulated in both OLK and OSCC when compared to normal tissue (Fig. S1a, d). Furthermore, the staining score increased with increasing dysplasia in OLK but decreased as differentiation occurred in OSCC while there was no difference in positive rate in the subtypes of OLK or OSCC (Fig. S1b, c, e, f).

**Table 1:** The relationship between clinical histopathological features of OLK and expression of PRC1

Factors	Number (*n*)	Positive rate	Intensity	Staining score	*p* value
Sex					
Male	14	53.43 ± 13.74	2.21 ± 0.58	111.93 ± 13.25	0.422
Female	10	52.80 ± 11.08	2.20 ± 0.42	116.80 ± 15.89	
Age (years)					
≤60	16	54.31 ± 13.85	2.19 ± 0.54	115.19 ± 15.18	0.563
>60	8	50.88 ± 9.45	2.25 ± 0.46	111.50 ± 12.91	
Location					
Buccal	8	53.5 ± 17.07	2.13 ± 0.64	110.25 ± 18.28	0.627
Tongue	10	51.50 ± 3.49	2.30 ± 0.48	114.60 ± 13.60	
Others	6	55.50 ± 8.55	2.17 ± 0.41	117.83 ± 9.85	
Dysplasia					
Without	5	57.60 ± 19.32	1.80 ± 0.45	96.80 ± 4.60	0.000***
Mild	7	48.57 ± 8.83	2.29 ± 0.49	107.71 ± 8.28	
Moderate	6	55.67 ± 7.66	2.17 ± 0.41	118.17 ± 5.81	
Severe	6	52.33 ± 14.29	2.50 ± 0.55	131.33 ± 8.55	

Expression of PRC1 in OLK was not relevant to their sex, age, and location of lesions (*p* *>* 0.05) while exhibited a positive correlation with the degree of epithelial dysplasia (*p* *=* 0.000). Error bars, mean ± SD (*n* ≥ 5); ***p < 0.001; *t*-test or Wilcoxon rank sum test.

**Table 2:** The association between clinical histopathological features of OSCC and expression of PRC1

Factors	Number (*n*)	Positive rate	Intensity	Staining score	*p* value
Sex					
Male	37	82.14 ± 8.23	2.68 ± 0.48	217.86 ± 33.48	0.881
Female	17	83.59 ± 7.57	2.64 ± 0.49	219.41 ± 34.75	
Age (years)					
≤60	31	83.18 ± 7.76	2.64 ± 0.49	218.18 ± 37.45	0.930
>60	23	82.55 ± 8.11	2.68 ± 0.48	219.09 ± 30.44	
Location					
Buccal	10	80.30 ± 8.59	2.80 ± 0.42	223.20 ± 33.62	0.936
Tongue	16	83.13 ± 8.19	2.60 ± 0.51	214.20 ± 35.46	
Gingival	12	82.25 ± 8.00	2.63 ± 0.52	214.38 ± 32.94	
Floor of oral	12	86.25 ± 7.82	2.63 ± 0.52	225.13 ± 35.59	
Others	4	82.67 ± 2.08	2.67 ± 0.58	219.67 ± 43.04	
Differentiation					
Well	31	83.74 ± 7.74	2.44 ± 0.51	202.41 ± 32.49	0.003*
Moderate	16	81.73 ± 7.12	2.87 ± 0.35	234.07 ± 27.29	
Poor	7	80.20 ± 9.66	3.00 ± 0.00	240.60 ± 28.96	
T stage					
T1	20	82.06 ± 8.21	2.41 ± 0.51	194.53 ± 25.52	0.003*
T2	10	82.60 ± 8.10	2.80 ± 0.42	230.20 ± 31.84	
T3	8	86.46 ± 5.94	2.55 ± 0.52	219.364 ± 39.45	
T4	16	79.56 ± 7.44	3.00 ± 0.00	239.67 ± 22.38	
*N* (lymph node metastasis)					
N0	28	82.41 ± 8.29	2.82 ± 0.40	231.50 ± 29.26	0.004*
N1N2	26	83.00 ± 7.27	2.48 ± 0.51	203.44 ± 33.49	
Clinical stage					
I/II	33	82.19 ± 7.74	2.55 ± 0.51	206.71 ± 31.39	0.005*
III/IV	21	83.75 ± 7.72	2.81 ± 0.40	235.69 ± 32.35	

No statistical difference between PRC1 and sex, age, tumor location was found in patients with OSCC. However, increased PRC1 was significantly associated with poor differentiation, large tumor volume, lymph node metastasis and high-clinical stage (*p* *<* 0.05). Error bars, mean ± SD (*n* ≥ 4); *p < 0.05; *t*-test or Wilcoxon rank sum test.

To explore the correlation between PRC1 and patients with OLK or OSCC, a method of correlation analysis was introduced to analyze. In this study, we found that the expression of PRC1 in OLK and OSCC was independent of patient sex, age, and location of lesions (*p* *>* 0.05) while there was a positive correlation with the degree of epithelial dysplasia in OLK (*p* *=* 0.000) (Table 1), being significantly associated with poor differentiation, large tumor volume, lymph node metastasis and high-clinical stage in OSCC (*p* *<* 0.05) (Table 2). Concomitantly, PRC1 expression as determined using immunostaining in those patients with local lymph node metastasis or at stage T2 was stronger than those at T1 or having no metastasis (*p* *<* 0.05). Interestingly, no statistical difference could be found between T2 and T3 (*p* *=* 0.499), T3 and T4 (*p* *=* 0.188) (Table 2).

**Fig. 2:**
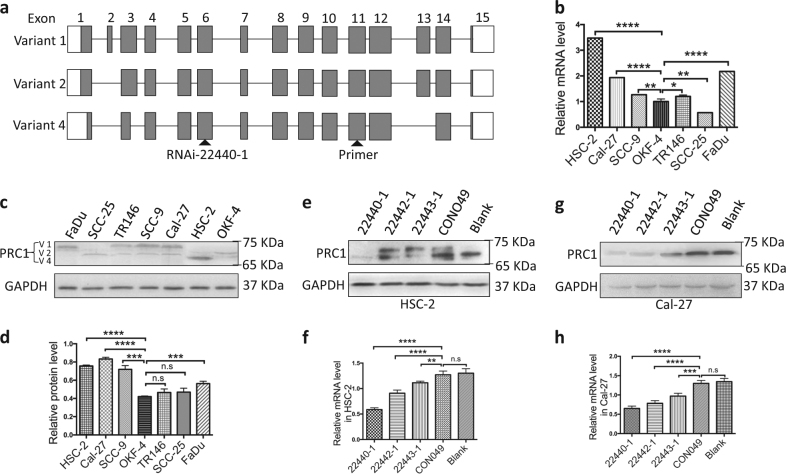
Genomic structure of PRC1, PRC1 expression in OSCC cell lines and its knockdown in HSC-2 and Cal-27 cells. **a** Genomic structure of PRC1 variants 1, 2, and 4. Gray boxes indicate protein-coding regions while white boxes indicate non-coding regions. RNAi-22440-1 locates in common exon 6 and primers in common exon 11. Genomic information was obtained from the NCBI database (V1: Gene, NM_003981.3; V2: Gene, NM_199413.2; V4: Gene, NM_001267580.1). **b** Higher PRC1 mRNA expression levels were observed in HSC-2, FaDu, and Cal-27 cells than other OSCC cell lines or OKF-4 cells. **c**, **d** PRC1 expression was high in HSC-2, Cal-27, and SCC-9 cells as observed using western blot analysis. **e**–**h** Combined RT-PCR and western blot analyses demonstrated that a PRC1-RNAi sequence (22440-1) can effectively downregulate PRC1 expression in HSC-2 and Cal-27 cells. GAPDH was used to normalize RT-PCR results, and as a loading control in western blots. All *n* = 3; error bars, mean ± SD; n.s, not significant, *p *<* 0.05, **p *<* 0.01, ***p *<* 0.001, ****p < 0.0001; *t*-test

Regarding its behavior in the seven cell lines, as shown in Fig. 1g–l, negative or weakly positive PRC1 staining was observed in OKF-4 cells while the staining in OSCC cell lines, including HSC-2, Cal-27, and SCC-9 (SCC-25 not shown), was positive. Combined with the results of mRNA (Fig. 2b) and protein (Fig. 2c, d), we found that HSC-2 and Cal-27 cells exhibited the highest expression of PRC1 not only at the protein level but also at the mRNA level among the 7 cell lines. Furthermore, HSC-2 cells exhibited the highest expression of “variant 4”, whereas Cal-27 cells and the majority of the other OSCC cell lines primarily expressed “variant 1”. Accordingly, HSC-2 and Cal-27 cell lines were mainly utilized in the subsequent experiments.

### Perturbation of PRC1 changed the biological properties of OSCC cell lines in vitro

**Fig. 3:**
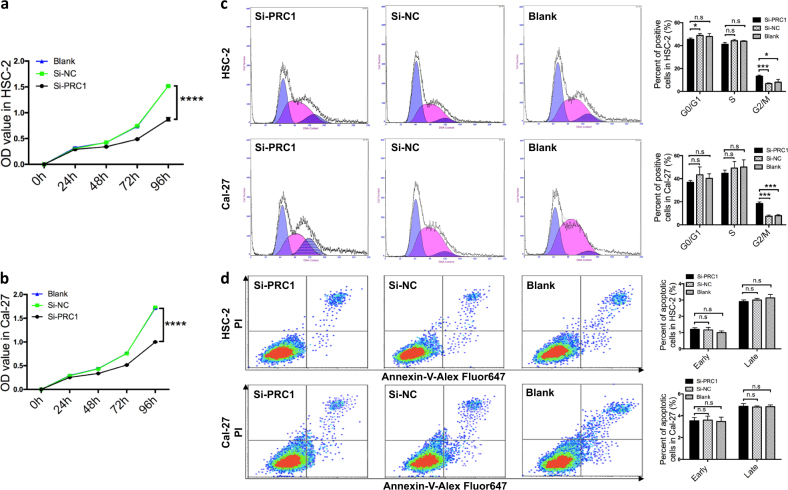
Roles of PRC1 knockdown in proliferation, cell cycle and apoptosis in vitro. **a**, **b** In the proliferation assay, the difference between si-PRC1-treated cells and control cells (si-NC and blank) was statistically significant after 48 h for Cal-27 and HSC-2 cells (*p*_Cal-27_ = 0.003 and *p*_HSC-2_ = 0.000). **c** HSC-2 and Cal-27 cells in the si-PRC1-treated group exhibited higher G2/M phase arrest after 72 h compared to controls. **d** No statistically significant difference in cell apoptosis was observed in either early or late stages 72 h after transfection. All *n* = 3; error bars, mean ± SD; n.s, not significant, *p < 0.05, **p < 0.01, ***p < 0.001, ****p < 0.0001; *t*-test

To assess the biological changes to OSCC resulting from the perturbation of PRC1, we performed further investigations. Firstly, we established two OSCC cell lines (HSC-2 and Cal-27) with downregulated PRC1. PRC1 si-RNAs (22440-1, 22442-1, and 22443-1) or si-NC (CON049) were transfected into cultured HSC-2 and Cal-27 cells respectively under the best infectious condition (ENi.S. +5 μg/ml Polybrene, multiplicity of infection = 80) resulting in a high transfection efficiency (83.7 ± 6.57%) (Fig. S2a). Based on RT-PCR data (Fig. 2f, h) and western blot analysis (Fig. 2e, g), we established that PRC1-RNAi (22440-1) was the most effective sequence. Following the decrease in expression of PRC1 in HSC-2 and Cal-27 cells, we further explored their cell biology. Flow cytometry (FCM) was conducted to analyze cell cycle, as shown in Fig. 3c. The cells were observed to be in G2/M phase arrest in the experimental group (si-PRC1) compared to si-NC or blank (*p* *<* 0.05). Similarly, a comparable situation was observed with Cal-27 cells (*p* *<* 0.001).

The role of PRC1 on proliferation was then evaluated. Using a CCK-8 analytic approach, as shown in Fig. 3a and b, a significantly lower rate of proliferation was observed in si-PRC1-treated cells compared with those transfected with the untargeted-control (si-NC) and blank after 48 h.

To understand whether PRC1 has a direct functional role in the regulation of cell death in OSCC, we performed FCM, trypan blue and propidium iodide (PI)/calcein fluorescence staining for further exploration. However, our study showed that no statistical difference was observed in the number of apoptotic HSC-2 or Cal-27 cells due to the intervention of si-PRC1 compared to si-NC or blank (Fig. 3d). Also, cell death in HSC-2 and Cal-27 cells could not be induced by PRC1 knockdown (Fig. S2b–g). Taken together, these data suggest that downregulation of PRC1 induces OSCC to stay in the G2/M phase, inhibiting cell proliferation, but has no clear effect on cell death.

### Downregulation of PRC1 inhibited tumor growth in vivo

**Fig. 4:**
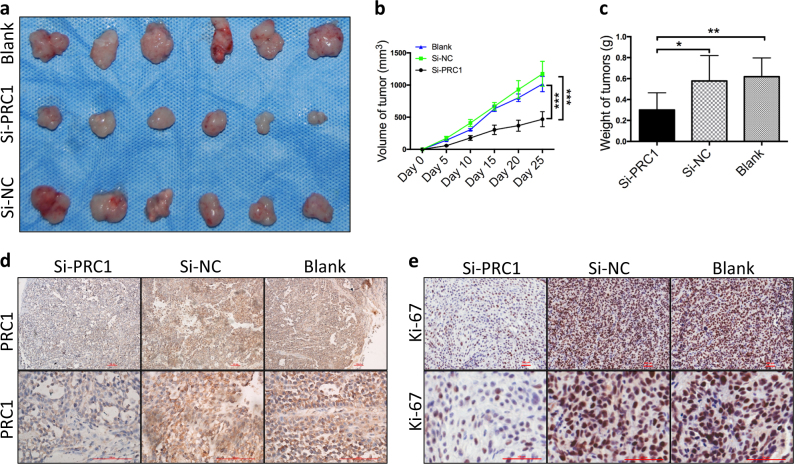
Downregulation of PRC1 inhibits tumor growth in vivo. **a** Gross tumors from nude mice. **b** Tumor volumes were smaller in the si-PRC1-treatment group than those from the untreated groups (si-NC and blank). **c** Weights of tumors derived from the si-PRC1-treated group were lower than those of the controls. **d** In the si-PRC1 group, tumors still exhibited PRC1 knockdown when compared to si-NC and blank groups as measured by IHC staining (scale bar = 100 μm). **e** Ki-67 located principally in the nuclei, its expression significantly decreased in the si-PRC1-treated group, as confirmed by IHC staining (scale bar = 100 μm). All *n* ≥ 3; error bars, mean ± SD; n.s, not significant, *p < 0.05, **p < 0.01, ***p < 0.001; *t*-test

After verifying that the expression behavior of PRC1 was closely related to proliferation and cell cycle in vitro, we continued to explore its effects on tumor growth. Totally, 18 nude mice were utilized in subcutaneous tumorigenicity assay. We found that the subcutaneous scleroma could be detected after inoculation in the si-NC and control groups on the 5^th^ day and after 7–10 days in the si-PRC1 group. As shown in Fig. 4a–c, tumors explanted from the si-PRC1-treated group had smaller volumes (468.30 ± 116.83 mm^3^) and their weights (0.3 ± 0.16 g) were lower than those from the untreated groups (si-NC and blank), differences that were statistically significant (*p* *<* 0.05). As judged by IHC, expression of PRC1 and Ki-67 was reduced significantly in the si-PRC1-perturbed group when compared to the control groups (Fig. 4d, e). Thus, these data indicate that reduced expression of PRC1 inhibits the tumor growth in vivo through suppression of cell proliferation.

### The related mechanism of PRC1 in the regulation of proliferation of OSCC

**Fig. 5:**
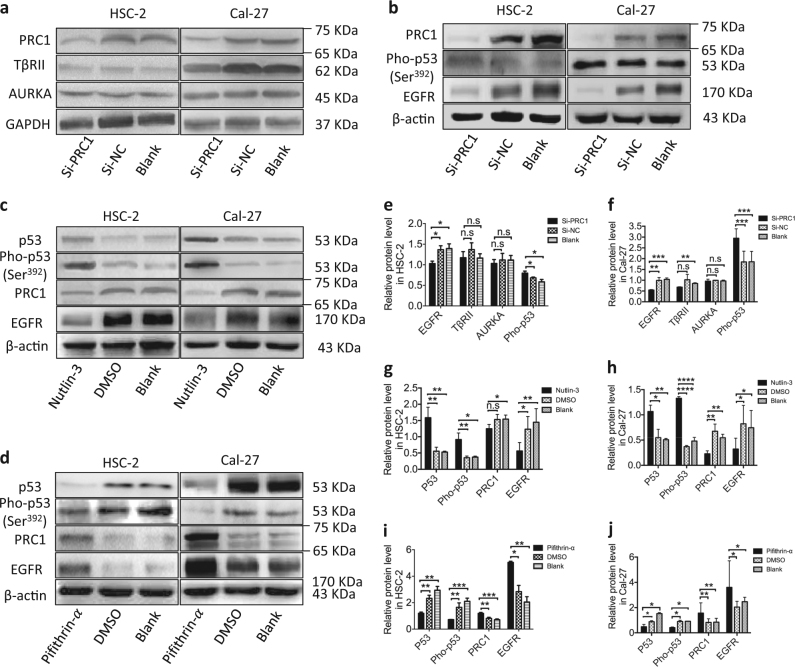
PRC1 knockdown activates p53 and inhibits the EGFR signaling pathway. **a**, **e**, **f** After PRC1 knockdown, TβRII and AURKA expression in HSC-2 cells and AURKA expression in Cal-27 cells were unaltered while TβRII was decreased in Cal-27 cells. **b**, **e**, **f** Expression of pho-p53 (Ser^392^) increased while EGFR decreased significantly following PRC1 knockdown. **c**, **g**, **h** Following activation with Nutlin-3, expression of both p53 and pho-p53 (Ser^392^) increased while that of PRC1 and EGFR decreased significantly. **d**, **i**, **j** P53 and pho-p53 (Ser^392^) were inhibited by Pifithrin-α, which in turn initiated an upregulation of both PRC1 and EGFR. All *n* *≥* 3*;* error bars, mean ± SD; n.s, not significant, *p *<* 0.05, **p *<* 0.01, ***p *<* 0.001, ****p *<* 0.0001; *t*-test

To understand the potential mechanism of PRC1 in cell proliferation, we measured the expression of epidermal growth factor receptor (EGFR), transforming growth factor β type II receptor (TβRII) and Aurora kinase A (AURKA), which are closely associated with proliferation and cell cycle, using western blot analysis. The expression of EGFR in HSC-2 cells was weaker than TβRII and AURKA in the si-PRC1-perturbed group (Fig. 5a, b, e, f). Unlike in HSC-2 cells, the protein levels of both EGFR and TβRII decreased in Cal-27 cells following PRC1 knockdown, while there was no significant change in AURKA expression compared with the control groups (Fig. 5a, b, e, f), suggesting that decreased EGFR levels following downregulation of PRC1 is a common event in OSCC.

Since PRC1 is a key target of p53, we evaluated the expression of PRC1 in HSC-2 and Cal-27 cells after both activation and inhibition of p53. Following activation by Nutlin-3, expression of both p53 and pho-p53 (Ser^392^) increased significantly in HSC-2 and Cal-27 cells while PRC1 decreased (Fig. 5c, g, h), indicating that p53 negatively affects PRC1 expression. Conversely, expression of PRC1 was increased by the downregulation of p53 in HSC-2 and Cal-27 cells treated with Pifithrin-α, an inhibitor of p53 (Fig. 5d, i, j).

## Discussion

In recent years, PRC1, a protein closely related to cytokinesis, has already been well documented for its structure and functions^14,16,20,21,23^. By alternative splicing into three variants (variant 1, variant 2 and variant 4), as shown in Fig. 2a and c, it can be considered a substrates of cyclin-dependent kinases, closely associated with the binding of microtubules and assembly of central spindles^14,16,23^. Previous studies have shown that knockdown or knockout of PRC1 is an efficient method of inhibition of tumor growth including breast, bladder and cervical cancers^15,18,24,25^. In this study, we devised a systems biology strategy that progressively cycled experiments and computations to predict and verify the expression of PRC1 in 7 cell lines and 95 clinical samples. Both the positive rate and staining score of PRC1 increased gradually in normal tissue, OLK and OSCC (Fig. S1a, d), indicating that PRC1 has a promoting role in oral tumorigenesis. Combined with the clinical data (Tables 1 and 2), we have provided evidence showing that a close correlation between PRC1 and OSCC progression exists, suggesting that PRC1 has a potential role in predicting malignancy and prognosis of OSCC.

Under normal conditions, presenting circular lines around cell nucleus at interkinesis, PRC1 locates in the chromosomal centromere at prophase then anchors with the mitotic spindle during metaphase and the first half of anaphase, aiming to inhibit elongation of the spindle^26^. It’s principle function is to assemble in the central spindle to recruit actin and kinases at telophase^26^. Our study suggests that knockdown PRC1 induces more HSC-2 or Cal-27 cells to enter G2/M phase arrest resulting in the termination of karyomitosis (Fig. 3c). Since PRC1 expresses highly in the G2 phase (Fig. 3c), it regulates elongation and polarity of the spindle^27,28^. Thus, its effective downregulation blocks the normal process of elongation and polarization of the spindle, causing G2/M phase arrest. These findings also suggest that it is a common event for PRC1’s involvement in regulating the cell cycle. The three variants of PRC1 might not have a different role in this process in OSCC cell lines since HSC-2 cells exhibit the highest expression of “variant 4”, whereas Cal-27 and the majority of the other OSCC cell lines primarily express “variant 1”.

Furthermore, G2/M cell cycle arrest could result in the inhibition of cell proliferation^1^. Previous studies have shown that some cancer cells are highly dependent on PRC1 for proliferation^29,30^. For instance, Shimo et al.^18^ found that intervention with siRNA against PRC1 in breast cancer cells effectively suppressed its expression and inhibited the proliferation of the cells. Similarly, our data also confirms this notion that downregulated PRC1 also inhibits tumor growth without significant toxicity to the cells (Fig. 4a, b, c).

In addition, interference of proliferative signaling is another appealing approach to inducing cell apoptosis in tumor cells since its proliferative response in normal cells while perturbed in most cancers^31–33^. In this study, we additionally performed FCM (Fig. 3d), and evaluated trypan blue and PI/calcein fluorescence staining (Fig. S2b–g) to investigate if downregulating PRC1 would induce any modification to cell death, including apoptosis in OSCC. However, we did not find that cell death in HSC-2 and Cal-27 cells was induced by PRC1 knockdown. Unlike the findings of Zhang et al.^34^ that PRC1 knockdown in two gastric carcinoma cell lines, AGS and HGC27, induced apoptosis, no significant difference in apoptosis was observed in HSC-2 and Cal-27 cells treated with si-PRC1 at prophase or telophase (Fig. 3d), demonstrating that the effect of PRC1 on cell apoptosis is highly tissue and tumor-specific. We propose an alternative hypothesis, that si-PRC1 might inhibit other signaling pathways such as Bcl-2 or Caspase, subsequently leading Cal-27 and HSC-2 cells to become resistant to cell death, including apoptosis, necrosis, and programmed cell death.

In vivo, we found that downregulation of PRC1 also inhibited the proliferation of OSCC which is in good agreement with the in vitro results (Figs. 3a, b and 4a–c). Furthermore, expression of PRC1 and Ki-67 decreased significantly in the si-PRC1-treated group compared to the untreated groups (Fig. 4d, e). Ki-67, a proliferating nuclear antigen during cell cycle phases G1, S, G2, and M, is closely related to karyomitosis in the process of proliferation^35,36^. Supporting our results, Dwivedi et al.^37^ also found that increased Ki-67 could be used to assess the severity of epithelial dysplasia and histological grading of OSCC. In sum, downregulation of PRC1 in OSCC could inhibit proliferation, subsequently leading to suppression of tumor growth in vivo.

**Fig. 6:**
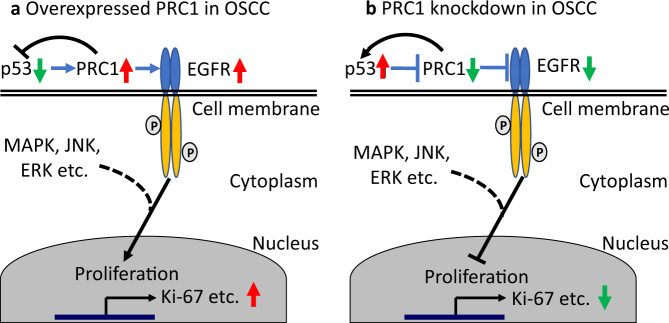
Schematic overview of p53/PRC1/EGFR in OSCC model. **a** In OSCC, inhibition of p53 leads to overexpression of PRC1, which in turn further suppresses p53. The EGFR signaling pathway is enhanced by overexpressed PRC1, subsequently leading to the promotion of proliferation. **b** After PRC1 knockdown in OSCC, p53 is upregulated which causes additional inhibition of PRC1 as feedback, which consequently suppresses the EGFR pathway and inhibits proliferation

Mechanistically, EGFR, TβRII, and AURKA related to the proliferation and cell cycle were included for the exploratory study^38–42^. The expression of EGFR was found to decrease, corresponding to PRC1 expression in the si-PRC1-treated group (Fig. 5b, e, f), indicating that PRC1 regulates EGFR signaling, consequently mediating cell proliferation. Since the p53 binding site locates in the EGFR promotor^43,44^, we further detected its expression at the protein level. Intriguingly, pho-p53 (Ser^392^) was attenuated by si-PRC1, suggesting that PRC1 exerts a negative effect on p53 (Fig. 5b, e, f). Furthermore, based on our data (Fig. 5c, g, h), we found that p53 and pho-p53 (Ser^392^) were both activated by Nutlin-3 which is a p53 activator, thereby leading to the downregulation of PRC1. Conversely, a decrease in p53 and pho-p53 (Ser^392^) caused by Pifithrin-α (a p53 inhibitor) augmented PRC1 expression (Fig. 5d, i, j). These results indicate that there is a loop between p53 and PRC1 (Fig. 6). Similarly, Liu et al.^21^ showed that p53 can directly inhibit the transcription of PRC1 with the result of regulating tumor cell mitosis and G2/M phase arrest. In addition, we found that the expression of EGFR changed in line with PRC1 resulting from treatment with Nutlin-3 or Pifithrin-α (Fig. 5c, d, g–j), further supporting the notion that PRC1 mediates the EGFR signaling pathway. Furthermore, the downstreams of EGFR pathway such as MAPK, JNK, ERK1/2, and so on exerting a regulation of proliferation in numerous tumors including oral cancer have been well documented^45–47^. In sum, these results suggest that the p53/PRC1/EGFR signaling axis is involved in the process of promoting cell proliferation, causing an abnormity of cell cycle transition in OSCC (Fig. 6).

Unlike the expression of EGFR regulated by PRC1 in HSC-2 and Cal-27 cell lines, we found that TβRII expression decreased in Cal-27 cells with no significant change in HSC-2 cells (Fig. 5a, e, f), illustrating that TβRII might exhibit paradoxical roles either as a suppressor or promotor of PRC1 in OSCC progression. An alternative hypothesis, supported by the variants of PRC1 in HSC-2 and Cal-27 cells, can be proposed, suggesting that variant 4 of PRC1 in HSC-2 cells may not be involved in regulating the expression of TβRII in OSCC. These findings suggest that the functional differences among these variants do exist and so utilization of PRC1 and its variants as an oral cancer therapy should be afforded more attention. With respect to AURKA, unlike the findings of Pan et al.^48^, who reported that it could destruct bipolar spindle structure, suppress tumor growth and induce cell apoptosis in OSCC, no significant change in AURKA expression was observed in our study (Fig. 5a, e, f), indicating it was not mediated by PRC1 in OSCC or other pathways, such as the co-binding of proteins that induces effects to those observed during mitosis.

In summary, based on our findings, for the first time, we have shown elevated expression of PRC1 in human OSCC tissues and an association with OSCC progression. Decreased expression of PRC1 suppresses cell proliferation and causes G2/M phase arrest but has no effect on apoptosis. Importantly, we have provided evidence that the p53/PRC1/EGFR signaling pathway is involved in the function of PRC1 in cell proliferation and cell cycle transition during tumor development in OSCC, highlighting the potential of PRC1 to become a novel target for OSCC treatment.

## Methods and materials

### Patients and tissue samples

In total, 95 samples from 54 patients with OSCC and 24 with OLK who underwent surgical resection and 17 normal individuals who received craniofacial plastic surgery or tooth extractions were obtained from the West China Hospital of Stomatology in Sichuan University. Tissue samples were immediately fixed in 10% buffered formalin and then embedded in paraffin prior to sectioning. The presence of cancer in tumor samples was confirmed by pathological examinations. This study was approved by the Human Research Ethics Committee of West China Hospital of Stomatology in Sichuan University (No. WCHSIRB-D-2012-0031-R1).

### Cell lines and culture conditions

A total of seven kinds of cell lines including HSC-2 (American Type Culture Collection [ATCC], Manassas, VA, USA), TR146 (European Collection of Authenticated Cell Cultures [ECACC], Porton Down, Salisbury, UK), SCC-25 (ATCC, Manassas, VA, USA), FaDu (ATCC, Manassas, VA, USA), SCC-9 (ATCC, Manassas, VA, USA), Cal-27 (State Key Laboratory of Oral Disease, Sichuan, China) and OKF-4/TERT-1 (Harvard Skin Disease Research Center, Brigham and Women’s Hospital, Boston, MA, USA) were included in this research. The anatomical sites of origin of these cell lines are as follows: oral cavity (HSC-2), tongue (SCC-9, SCC-25 and Cal-27), floor of mouth (OKF-4/TERT-1) and hypopharynx (FaDu and TR146). HSC-2, TR146, FaDu and Cal-27 cells were maintained in Dulbecco’s modified Eagle’s medium (DMEM) (Gibco, Grand Island, NY, USA). SCC-9 and SCC-25 cells were cultured in a 1:1 mixture of DMEM/F-12 medium. OKF-4/TERT-1 cells were cultivated in keratinocyte or Ham’s F-12 culture medium. Each base medium was supplemented with 10% fetal bovine serum (Gibco, Grand Island, NY, USA). Cells were cultured at 37 °C in a humidified atmosphere with 5% CO_2_.

### Immunohistochemistry (IHC) and immunocytochemistry (ICC)

Human and mouse tumors were dissected and immediately fixed in 10% buffered formalin then embedded in paraffin. Paraffin-embedded tissues were sliced into 5 μm-thick sections then used for H&E or immunohistochemical staining. For immunocytochemistry, cells were seeded on glass coverslips at a density of 2.0 × 10^4^/cm^2^ then cultured to about 60% confluence prior to fixation and permeabilization in 10% buffered formalin and 0.5% Triton X-100. Cells were stained using the SP-9000 Histostain^TM^-Plus Kits (BIOZ, Porter Drive Palo Alto, CA, USA) according to the manufacturer’s instructions using antibodies against PRC1 (1:150, Abcam, Cambridge, UK) and Ki-67 (1:200, ImmunoWay, Plano, TX, USA) were. Specific antigens in ten randomly-selected fields were visualized by two independent investigators using a light microscope at a power of ×400, as per the method of Kreisberg et al. ^49^

### **p53 interference**

Cells of 1 × 10^4^ per well were seeded into the 6-well plates and incubated at 37 °C for 24 h. Then, 0.1% Nutlin-3 (Sigma-Aldrich, St Louis, MO, USA) dissolved in dimethyl sulfoxide (DMSO) (MP Biomedicals, Santa Ana, CA, USA) to a concentration of 25 μM for Cal-27 and 20 μM for HSC-2 was added into the cells respectively. Identically, 0.1% Pifithrin-α (Beyotime, Shanghai, China) dissolved in DMSO to a concentration of 35 μM for Cal-27 and HSC-2 cells was added. After 48 h, the total protein was extracted for western blot.

### Quantitative real-time PCR (qRT-PCR)

For gene-detection, total RNA was isolated from cells using Trizol reagent (Invitrogen, Grand Island, NY, USA) and quantified by measuring the absorbance at 260 nm. First-strand cDNA was synthesized from total RNA using a Perfect Real Time PrimeScript® RT reagent kit (Takara, Shiga, Japan), according to the manufacturer’s protocol. The cDNA was quantified by polymerase chain reaction (PCR) on a 7300HT Real-Time PCR System using SYBR® Premix Ex TaqTM II (Takara, Shiga, Japan). GAPDH was used as the endogenous control. The PCR primer pairs were synthesized as follows: PRC1 sense: 5′-TAGACCACACCCCAGACACAAG-3′ and antisense: 5′-CCCCTCACACACTGCTTCATT-3′; GAPDH sense: 5′-CTTTGGTATCGTGGAAGGACTC-3′ and antisense: 5′-GTAGAGGCAGGGATGATGTTCT-3′. All the procedures were performed according to the manufacturer’s instructions. Additionally, the comparative CT method (ΔΔCT method) was used to quantify target gene expression in comparison with the control.

### Western blot analysis

Whole-cell protein extracts were prepared using a lysis buffer containing phosphatase inhibitor, protease inhibitor and pheylmethylsulfonyl fluoride (KeyGEN Bio-TECH, Jiangsu, China). For western blot analysis, proteins were subjected to 10 and 5% SDS-PAGE (Beyotime, Shanghai, China) using standard techniques. After transferring to the PVDF membrane (Millipore, Darmstadt, Germany), the protein contents were probed with anti-EGFR, TβRII, AURKA and p53, pho-p53 (Ser^392^) (1:500, ImmunoWay, Plano, TX, USA), and anti-PRC1 (1:5000, Abcam, Cambridge, UK) antibodies, using GAPDH as a control. Horseradish peroxidase-conjugated secondary antibodies were reacted with the primary antibodies above. Proteins were visualized using an electrochemiluminescence system (Pierce Biotech, Grand Island, NY, USA).

### Lentiviral construction and cell transfection

Recombinant lentivirus vectors were purchased from Shanghai Genechem Co., Ltd (http://www.genechem.com.cn). Targeted sequences for shRNA PRC1 (shPRC1) were designed as follows: (1). PRC1-RNAi (22440-1): 5′-GGAACATTCAAAGGCATTT-3′; (2). PRC1-RNAi (22442-1): 5′-GCTGCAATTAGAAGTGGAT-3′; (3). PRC1-RNAi (22443-1): 5′-AGCTTTGTTAAATTGTGTT-3′. Recombinant viruses were generated using 293T packaging cells and a U6-MCS-Ubi-EGFP vector. Specific and control viruses were denoted si-PRC1 and negative control lentivirus (si-NC), respectively. Subsequently, si-PRC1 and si-NC were transfected into HSC-2 and Cal-27 cells and cultured in six-well plates for 12 h. The supernatant was removed and replaced with fresh culture medium containing 10% FBS. The cells were harvested for qRT-PCR and western blot analysis 72–96 h after transfection. When the infected cells exhibited a mean 80% decrease in PRC1 expression, they were used in the following experiments.

### Cell cycle analysis

The distribution of cells in the cell cycle was evaluated by FCM. After seeding the cells at a density of 5.0 × 10^6^ cells/petri dish (100 mm diameter), they were incubated in DMEM without serum for 24 h and then replaced by DMEM containing 10% FBS for 72 h. The cells were then trypsinized, resuspended in 70% ethanol, washed and incubated in phosphate-buffered saline (PBS) containing PI and RNAse A (KeyGEN Bio-TECH, Jiangsu, China) for 30 min at 37 °C. DNA contents were then analyzed using an Elite ESP flow cytometry (Beckman Coulter, CA, USA).

### Cell proliferation assay

A cell counting kit-8 (CCK-8, Dojindo, Japan) assay was used to quantify cell metabolism by measuring optical density (OD) of HSC-2 and Cal-27 cells after treatment in si-PRC1, si-NC and blank. Cells were firstly cultivated in a 96-well plate at a concentration of 1.0 × 10^3^ per well, and then incubated at 37 °C for 24, 48, 72 and 96 h. For each time point, the OD at 450 nm was measured using a spectrophotometer (Thermo, Grand Island, NY, USA) 2 h after adding CCK-8, according to the manufacturer’s instructions. All tests were performed in triplicate.

### Measurement of cell death

HSC-2 and Cal-27 cells were transfected with either si-PRC1 or si-NC, respectively. After 72 h post-transfection, detached cells were collected from the medium by centrifugation (300×*g*, 4 °C, 5 min) while those that were adherent were harvested by trypsinization without ethylenediaminetetraacetic acid. For the apoptosis assay, 1.0 × 10^5^ cells were resuspended in binding buffer (100 μl) containing 5 μl Annexin V-Alexa Fluor 647 and 10 μl PI for 15 min in the dark at room temperature. After adding 400 μl PBS, cell apoptosis was detected using an Elite ESP flow cytometer (Beckman Coulter, CA, USA). Annexin V-positive cells were considered apoptotic. Trypan blue (Beyotime, Shanghai, China) and PI/calcein fluorescence staining (KeyGEN Bio-TECH, Jiangsu, China) were performed according to the manufacturer’s instructions.

### Tumorigenesis assay in nude mice

All experiments involving animals were conducted in accordance with the U.S. Public Health Service’s policy on the humane care and use of laboratory animals. Eighteen 4-week-old BALB/c athymic nude mice were used, half being males and half females, purchased from the State Key Laboratory of Biotherapy and Cancer Center at Sichuan University. They were randomly divided into the three groups: negative control, si-PRC1 or si-NC. PRC1 mimic-transfected HSC-2 cells (5.0 × 10^5^) were suspended in 0.1 ml PBS and then injected subcutaneously into the dorsal of nude mice. Tumor major and minor axes of each tumor were measured every 5 days using calipers from day 5 following injection. Tumor volume was calculated as follows: tumor volume = 0.52 × (major axis) × (minor axis)^2^^50^. On the 25th day after injection, all mice were killed using luxation and the tumors were excised and weighed. The expression of Ki-67 and PRC1 in tumor samples was mainly detected by IHC.

## Electronic supplementary material

Supplementary figure S1

Supplementary figure S2

Supplementary figure legends
