# Supplementary figures and images for: Regulation of proliferation and cell cycle by protein regulator of cytokinesis 1 in oral squamous cell carcinoma
Source: Cell Death Dis. 2018 May 11;9(5):564. doi: 10.1038/s41419-018-0618-6 (PMC5948203; doi:10.1038/s41419-018-0618-6)

## Slide 1
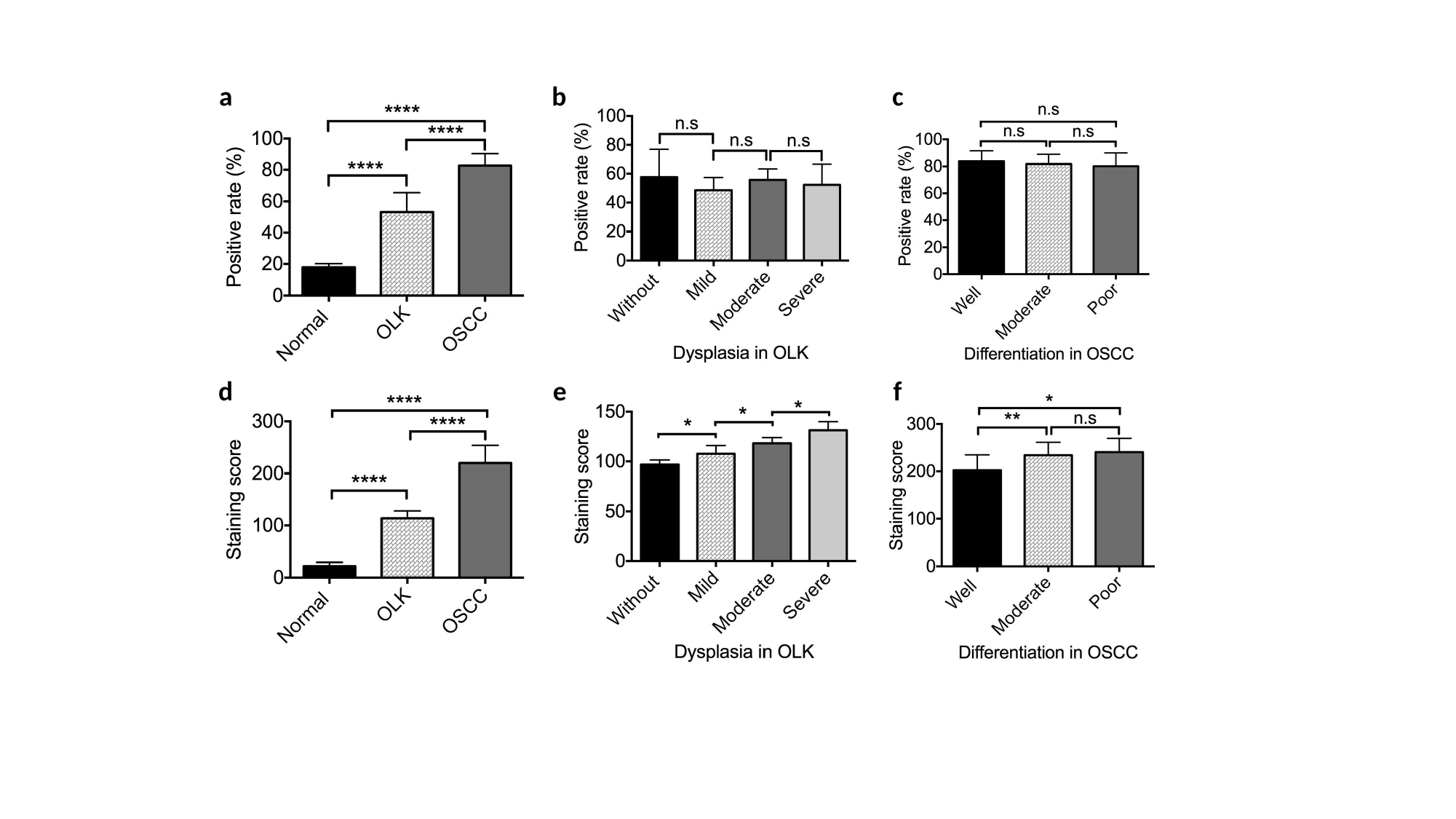

a
b
c
e
f
d

Supplement: Supplementary file 1 — Supplementary figure S1 [file 41419_2018_618_MOESM1_ESM.pptx]

## Slide 1
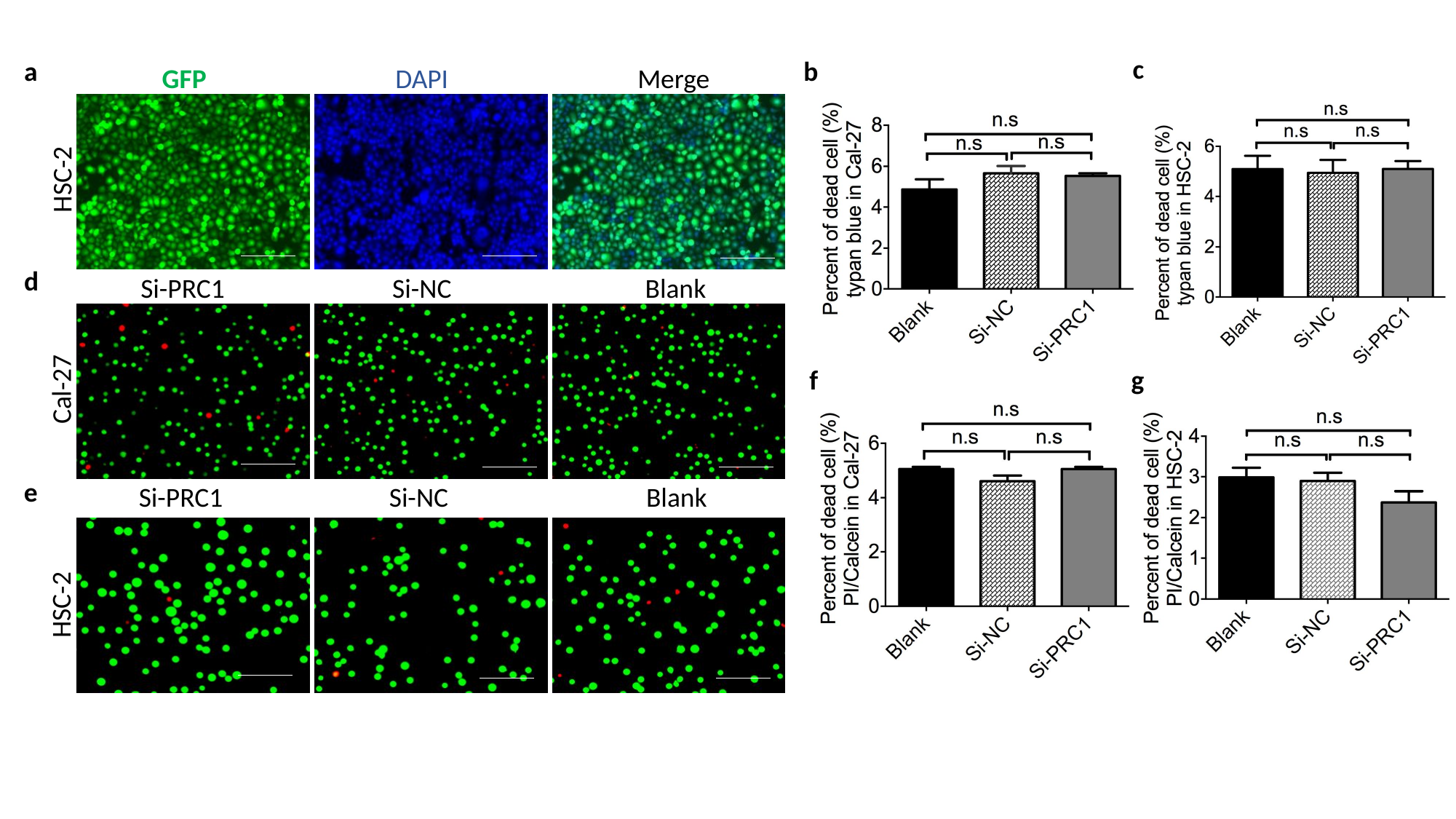

c
a
b
GFP
DAPI
Merge
HSC-2
d
Si-PRC1
Si-NC
Blank
f
g
Cal-27
e
Si-PRC1
Si-NC
Blank
HSC-2

Supplement: Supplementary file 2 — Supplementary figure S2 [file 41419_2018_618_MOESM2_ESM.pptx]
