# Supplementary material for: Regulation of proliferation and cell cycle by protein regulator of cytokinesis 1 in oral squamous cell carcinoma
Source: Cell Death Dis. 2018 May 11;9(5):564. doi: 10.1038/s41419-018-0618-6 (PMC5948203; doi:10.1038/s41419-018-0618-6)
Supplement: Supplementary file 3 — Supplementary figure legends [file 41419_2018_618_MOESM3_ESM.docx]

**Legends for supplementary figures**

**Fig. S1:**

**Different expression of PRC1 in normal tissue, OLK and OSCC. a** The positive rate of PRC1 increased gradually in normal tissue, OLK and OSCC. **b-c** No clear difference in positive rate could be found in the subtypes of OLK or OSCC. **d** The staining score of PRC1 increased gradually in normal tissue, OLK and OSCC. **e-f** The staining score increased with increasing dysplasia in OLK but reduced as differentiation occurred in OSCC. All *n* ≥ 5*;* error bars, mean ± SD; n.s, not significant, **p* < 0.05, ***p* < 0.01, ****p* < 0.001, *****p* < 0.0001; t-test.

**Fig. S2:**

**Transfection of PRC1 in vitro and its downregulation in cell death. a** GFP (green) indicates that the cells were successfully transfected by lentivirus for shPRC1, DAPI (blue) stains the nuclei (scale bar = 50 μm). **b-c** As observed using a trypan blue assay, no difference in cell death was observed in HSC-2 and Cal-27 cells in the si-PRC1 group. **d-g** No clear difference was found among the groups following PI/calcein fluorescence staining in Cal-27 and HSC-2 cells, living cell (green), dead cell (red) (scale bar = 50 μm). All *n* = 3; error bars, mean ± SD; n.s, not significant; t-test.
